# Rapid instructed task learning (but not automatic effects of instructions) is influenced by working memory load

**DOI:** 10.1371/journal.pone.0217681

**Published:** 2019-06-06

**Authors:** Maayan Pereg, Nachshon Meiran

**Affiliations:** Department of Psychology and Zlotowski Center for Neuroscience, Ben-Gurion University of the Negev, Beer-Sheva, Israel; Baycrest Health Sciences, CANADA

## Abstract

The ability to efficiently perform actions immediately following instructions and without prior practice has previously been termed Rapid Instructed Task Learning (RITL). In addition, it was found that instructions are so powerful that they can produce automatic effects, reflected in activation of the instructions in an inappropriate task context. RITL is hypothesized to rely on limited working memory (WM) resources for holding not-yet implemented task rules. Similarly, automatic effects of instructions presumably reflect the operation of task rules kept in WM. Therefore, both were predicted to be influenced by WM load. However, while the involvement of WM in RITL is implicated from prior studies, evidence regarding WM involvement in instructions-based automaticity is mixed. In the current study, we manipulated WM load by increasing the number of novel task rules to be held in WM towards performance in the NEXT paradigm. In this task, participants performed a series of *novel* tasks presented in mini-blocks, each comprising a) instructions of novel task rules; b) a NEXT phase measuring the automatic activation of these instructed rules, in which participants advance the screen using a key-press; and c) a GO phase in which the new rules are first implemented and RITL is measured. In three experiments, we show a dissociation: While RITL (rule implementation) was impaired by increased WM load, the automatic effects of instructions were not robustly influenced by WM load. Theoretical implications are discussed.

## Introduction

In the past few years, there is growing interest in the human ability to perform actions immediately following instructions and without prior practice [1–3]. This ability is mostly evident in relatively simple tasks that combine a small number of familiar elements in a novel association and is termed Rapid Instructed Task Learning (RITL; [4]). RITL can be exemplified in many real-life situations, such as operating a machine or software for the first time, or assembling a new piece of furniture. Moreover, instructions are sometimes so powerful that they even produce automaticity, reflected in activation of the newly instructed rules in an inappropriate context [2,5], henceforth “automatic effects of instructions”. Since the *instructed* task-rules have never been executed before, their representations (at least in their first implementation) cannot rely on long-term memory (LTM) traces from past performance [6,7]. Thus, instructions-based performance was hypothesized to rely on active maintenance in WM [1,8,9], a neuro-cognitive system responsible for holding *novel* representations [10].

In the following Introduction, we first review the theoretical basis and empirical evidence for the involvement of WM in RITL in order to establish the hypotheses for the current study. Next, we review the theoretical and empirical basis for our hypotheses concerning WM involvement in automatic effects of instructions.

### Theoretical involvement of working memory in rapid instructed task learning

Extant models assume that WM has limited resources, and that it interacts with LTM, as well. For example, Cowan [11] characterized WM as comprising of both a central component, severely limited in resources, and a comprehensive component relying on LTM and characterized by a much higher availability of resources. Oberauer’s [12] WM model may be viewed as an elaboration of Cowan’s model. It divides WM into two parts: procedural and declarative. The procedural subsystem is responsible for holding novel bindings between stimuli and responses; whereas the declarative subsystem is responsible for holding novel bindings between stimulus elements. Each of these subsystems is divided into three components: a) “activated LTM" that has a very large capacity, and can make familiar representations highly accessible; b) "bridge" (or “region of direct access” in the declarative system), with severely limited capacity, holding novel bindings between familiar representations (e.g., a novel binding between a familiar stimulus and a familiar response, i.e., a novel *task-rule*); and c) "response focus" (or “focus of attention” in the declarative system), which only holds the representation of the currently selected item/task-rule.

The current study does not aim to contribute to the debate regarding whether the distinction between the procedural and declarative WM subsystems is justified [13–16]. We simply note that, since RITL focuses on instructed task rules which are to be executed, we refer to the information regarding the instructions as *procedural* information. In detail, when a task rule is novel and has never been executed before, the familiar elements of the rule can be represented in activated LTM, but their novel elements (e.g., the novel binding between a stimulus and a response; S-R binding) must be represented in the “bridge”, which is a part of procedural WM in Oberauer’s model. This point is exemplified below.

**Fig 1:**
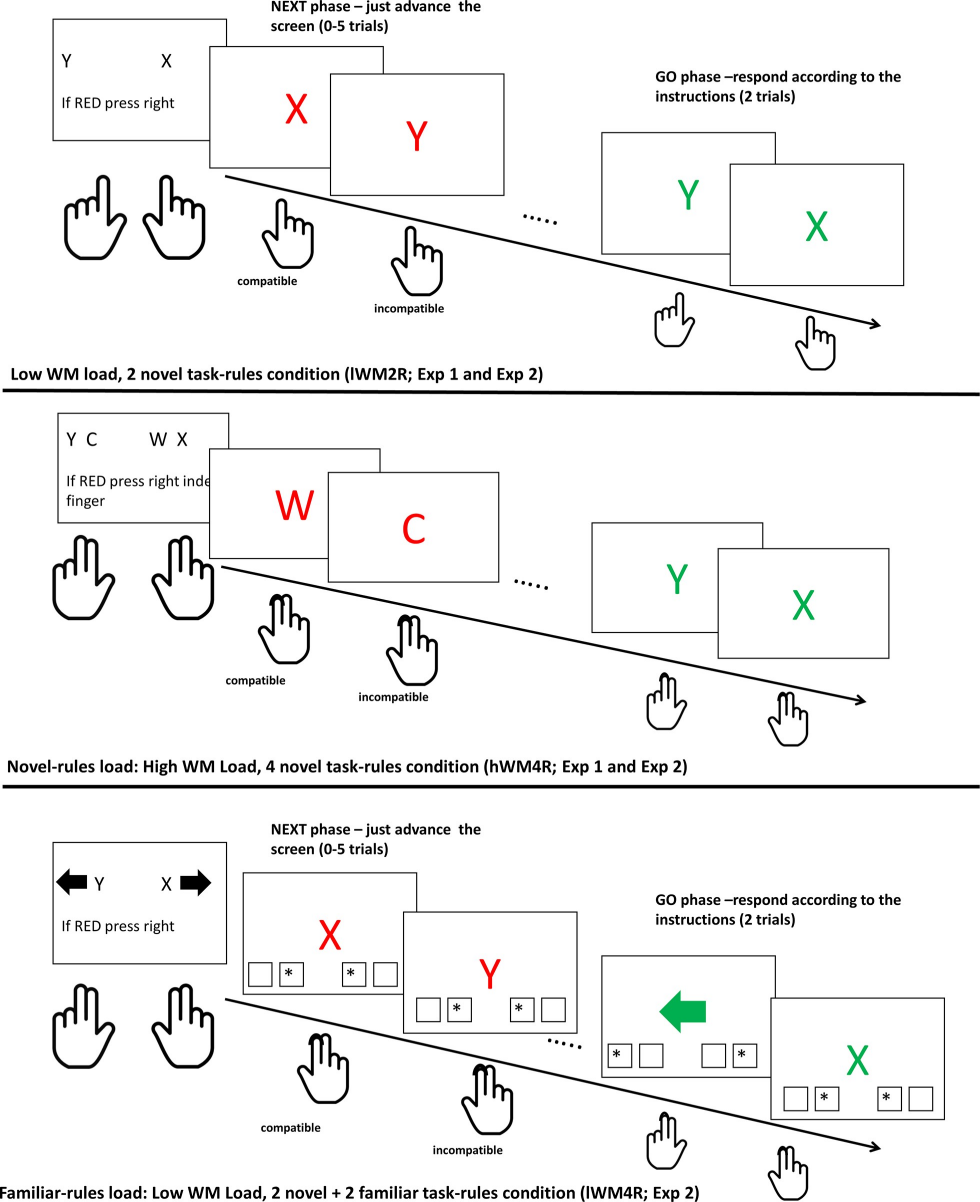
Mini-block structure, Experiments 1 and 2. Upper panel: low WM load, 2 novel task-rules; middle panel: novel-rules load, high WM load with 4 novel task-rules (Experiment 1 and Experiment 2); lower panel: familiar-rules load, low WM load with 2 novel task-rules + 2 familiar task-rules (Experiment 2). In all conditions of Experiment 2, the marked squares at the bottom of the screen (denoting the possible responses) appeared as shown in the lower panel.

In order to illustrate the involvement of WM in RITL tasks, let us consider the NEXT paradigm [5], which was used in the current study. In this paradigm, participants encounter a series of *novel* choice tasks, each comprising two S-R bindings (e.g., “Y” → press left, and “X” → press right; see Fig 1, upper panel). Each such novel task is implemented twice during a GO phase where the targets appear in GREEN color. How is each such novel task represented? In this example, the target stimuli “X” and “Y”, as well as the responses “right” and “left” are familiar elements and as such, they are represented in activated LTM. However, the binding of stimuli to responses is novel and is therefore thought to be represented in the “bridge” of procedural WM. This situation holds true at least until task implementation begins and LTM traces start to form [13]. In other words, Oberauer’s model seems to imply that RITL depends on (procedural) WM. In the following section, we review studies supporting the hypothesized involvement of WM in RITL.

### Empirical evidence concerning the involvement of working memory in rapid instructed task learning

Gathercole, Durling, Evans, Jeffcock, & Stone [17], who studied relatively complex tasks, found that children who were better able to perform novel oral instructions had relatively high WM capacity. Yang et al. [9] additionally showed that young adults’ accuracy of recalling/enacting instructions decreased in conditions that compromised WM functions (i.e., increasing WM load). In contrast, in an individual differences study using the NEXT paradigm, Meiran, Pereg, Givon, Danieli, and Shahar [18] showed that RITL was positively correlated with a factor of general-fluid intelligence, and with performance in 6-choice tasks involving *novel*-arbitrary bindings (in which the S-R rule had never been implemented beforehand, thus presumably tapping procedural WM). However, RITL was equally strongly correlated with performance in similar 6-choice tasks involving *familiar* bindings (in which the S-R rule is well-practiced, hence barely tapping procedural WM). This last finding questions the unique involvement of WM in the aforementioned individual-differences correlations. Moreover, RITL was not significantly correlated with WM capacity, as measured with complex-span tests. To account for these surprising findings, Meiran et al. suggested that perhaps the low task-rule set-size involved in the NEXT paradigm (only two task rules) was not large enough to challenge (and thus assess) WM-related individual differences.

Interestingly, Ruge et al. [19] used a larger task-rule set-size of four S-R rules (and thus presumably complex-enough to be sensitive to WM capacity differences), and manipulated WM load as well. They found that instruction-based learning was generally influenced by WM load, such that performance was worse under a WM-demanding condition. However, similar to Meiran et al. [18], Ruge et al. also did not observe significant correlations between instructions-based learning and individual differences in WM capacity (measured with simple-span tasks); albeit using a RITL task with an increased rule set-size.

To summarize, it seems that in accordance with the theoretical role of WM in keeping novel information, increasing WM load is likely to impair RITL performance. However, there are also indications regarding more complex regularities concerning individual differences in WM capacity. Given that individual differences were not measured in the current study, we predicted that WM load would impair RITL performance, confirming prior evidence [9,19].

In the following section we review theoretical and empirical evidence regarding an automatic by-product of RITL that could be measured just prior to the implementation of the new task.

### Involvement of working memory in automatic effects of instructions

Automatic effects of instructions reflect the case in which novel instructions are prematurely activated in an inappropriate context [2,20,21]. Specifically, and going back to the NEXT paradigm, just prior to implementing the instructions (in the GO phase), participants encounter the instructed stimuli in RED color, where they are requested to simply advance the screen using a constant NEXT response (either left or right, counterbalanced between participants; see Fig 1, upper panel). Advancing the NEXT targets using a response that is part of the instructed S-R set creates compatibility relations. For example, if a participant was instructed to press “left” in response to a target “Y” (as seen in the upper panel of Fig 1), and was also instructed to use the right key for NEXT responses, then responding “right” in response to a RED “Y” is considered incompatible with the RITL instructions. Concomitantly, responding “right” in response to a RED “X” is considered compatible with the RITL instructions. Indeed, results from a number of studies demonstrate the NEXT compatibility effect, showing that incompatible NEXT responses are slower and less accurate relative to compatible NEXT responses [5,18,22]. Importantly, during the NEXT phase, the novel instructions are not yet implemented, and are thus thought to be stored in a pending state in WM.

Given that the instructions are assumed to be held in WM during the NEXT phase towards execution in the GO phase, it is reasonable to predict that the automatic effect of instructions (seen in NEXT phase performance) might also be influenced by WM load. This prediction has already been tested by Cohen-Kdoshay and Meiran [23,24] who focused on another index for the automatic effects of instructions: the first-trial flanker compatibility effect.

In their study, Cohen-Kdoshay and Meiran administered a modified flanker paradigm [25] in which participants learned a novel category-response rule (e.g., beginning of the alphabet→right; end of the alphabet→left). In each trial, a target stimulus appeared in the middle of the screen (e.g., “A”) accompanied by flankers, which were visually different, but belonged either to the same response category (e.g., “BAB”, all belonging to the beginning of the alphabet) or to the alternative response category (e.g., “ZAZ”, target belonging to the beginning of the alphabet and flankers belonging to the end of the alphabet). Unlike in standard flanker tasks, where the first trials after the instructions are discarded, Cohen-Kdoshay and Meiran focused on these trials, and found that participants were slower when the flankers were incompatible than when they were compatible. Since this “first-trial flanker effect” was found immediately following the instructions, without prior practice, it was considered to reflect an automatic effect of instructions. Cohen-Kdoshay and Meiran [23] and Meiran & Cohen-Kdoshay [26] further tested the involvement of WM in this effect, by using a WM load manipulation involving a secondary go-no-go task that changed in every block. Their results showed that the first-trial flanker effect was eliminated under WM load. These authors additionally showed that the elimination of the effects was *not* due to the multitasking requirement of the load task, since the first-trial flanker effect was not eliminated when the secondary task was pre-trained and, hence, based on information already stored in LTM.

Nonetheless, Cohen-Kdoshay and Meiran’s conclusions are open to alternative explanations, since it is possible that the effect actually reflects semantic priming (see Neely [27] for a review) of the target stimulus by its flankers. For example, in the target stimulus “BAB”, the flankers prime the semantic category "beginning of the alphabet" and thus facilitate the encoding of “A”. Importantly, the *elimination* of the first-trial flanker effect by WM load could also be explained in terms of semantic priming, since it has been shown that WM load may reduce semantic priming effects [28]. Accordingly, the lack of instructions-based automaticity under WM load in Meiran and Cohen-Kdoshay’s [26] study could reflect the WM load-related attenuation of (LTM-based) semantic priming, rather than of (WM-based) automatic effects of instructions.

If the first-trial flanker compatibility effect studied by Meiran and Cohen-Kdoshay [23,24,26] truly reflects an automatic effect of instructions, then it follows that the NEXT compatibility effect described above would also reduce, or even be eliminated, under WM-load. An opposite prediction can be made on the basis of the individual differences study by Meiran et al. [18], who found *reversed* effects concerning the instructions-based automaticity: While WM capacity was not directly correlated with the NEXT compatibility effect, it was found that better performance in choice tasks was correlated with *small* NEXT compatibility effects. Assuming that loading WM is analogous to having poor WM, these results lead to the prediction that WM load should *enhance*, rather than reduce, the NEXT compatibility effect.

A third option comes from recent results regarding the influence of (procedural) WM training on RITL [29], that show a dissociation between the GO and NEXT phases. Specifically, it was found that participants who went through a rather extensive WM training were relatively efficient in their GO phase performance (i.e., RITL), whereas their NEXT compatibility effect was comparable to the control groups who were trained in tasks with little WM involvement or were not trained at all. Theoretically, such a dissociation could be supported by a recent model [8], as will be further elaborated on in the General Discussion.

To sum up, contrary to RITL, where relatively clear predictions could be made, the evidence regarding automatic effects of instructions lead to far less clear predictions. The results from the current study thus have the potential to shed light on this issue, especially due to the fact that this is the first study that employs an experimental WM load manipulation in a task that assesses both RITL and automatic effects of instructions.

### The current study

In three experiments, we aimed to test the involvement of WM in RITL and in the automatic effects of instructions, using the NEXT paradigm [5]. We followed Cole et al. [8], who propose that the limits of RITL might be tested by overflowing WM capacity (e.g., manipulating WM load). Our means to overload WM was by adding novel task-rules to the task. Based on Shahar et al. [16], who showed that WM in choice-tasks was influenced by the number of *novel* task-rules (but not familiar ones), manipulating WM load was done by increasing the number of novel task-rules. Experiment 1 tested whether this WM load manipulation influenced RITL and instructions-based automaticity; and Experiments 2 and 3 tested the influence of increased number of task-rules that either tap WM (novel arbitrary rules) or not (familiar non-arbitrary rules).

Importantly, both theoretically and empirically, the first implementation in RITL is considered to be the most important one [5,8]. In addition, the first-trial compatibility effect (measuring automatic effects of instructions) was found to be larger relative to more advanced trials [5,24]. Therefore, we chose to analyze the results in such a way that would allow us to reach conclusions regarding the influence of WM load on specific trials (see the Data analyses section for an elaboration).

The predictions regarding the influence of WM on RITL were quite clear: We hypothesized that RITL (GO phase performance) should be impaired under conditions characterized by a high WM load (i.e., increased number of *novel* task-rules), relative to when WM load is lower (i.e., a low number of novel task-rules, and increased number of *familiar* task-rules). However, given the mixed evidence regarding WM and automatic effects of instructions, the related predictions were less clear cut and patterns of increase, decrease, and no-change due to WM load all seemed plausible.

## Experiment 1

Following Shahar et al. [16], we loaded WM by manipulating novel-rules load. To do so, we increased the number of novel arbitrary task-rules from two task-rules (as in the original NEXT paradigm [5]), to four task-rules. We directly compared low and high WM-load conditions (i.e., 2 vs. 4 novel task-rules) in a *within-subjects* design.

### Method

#### Participants

All experiments in this study were approved by the Psychology department’s ethical committee. Twenty-two Ben-Gurion University of the Negev undergraduate students (19 women, *M*_age_ = 22.86, *SD* = 1.01) participated in the experiment for course credit. The sample size was originally determined based on a power analysis using G-Power 3.1.9.2 [30] that was set to obtain an effect size equivalent to Ƞ^2^_p_ = 0.09 with a power of 80%. All of the participants signed an informed consent form, reported having normal or corrected-to-normal vision, including intact color vision, and not having been diagnosed for attention deficits.

#### Materials and procedure

We adapted the NEXT paradigm used in Meiran et al. [5], Experiment 1. We defined two conditions: the first including two novel task-rules was hypothesized to involve low WM load; and the second including four novel task-rules was hypothesized to involve high WM load. The experiment consisted of 72 mini-blocks: 36 with a low WM load (2 novel task-rules; Fig 1, upper and middle panels) and 36 with a high WM load (4 novel task-rules).

In each mini-block, 2 or 4 stimuli were mapped to 2 or 4 key-press responses, respectively, towards a short execution during a GO phase (in which the target stimuli appeared in GREEN color). Participants were requested to press the spacebar once they were ready to perform the task, but only after at least 3 seconds had elapsed (this feature was added to ensure that participants do not mistakenly skip the instructions). Just prior to execution, participants encountered the stimuli in RED color during a NEXT phase, in which they were requested to simply advance the screen using either their right (or left) index finger (i.e., the K or S keys, respectively, counterbalanced between participants). The number of NEXT trials was pseudo-exponentially adjusted to be between 0–5 trials, in order to discourage expectation of the *timing* of the upcoming GO phase. Approximately 10% of the mini-blocks did not include a NEXT phase (i.e., 0 NEXT trials), with the intention to promote high readiness towards the GO task's performance immediately after the instructions. (For further details regarding the experimental procedure, refer to Meiran et al., [5], Experiment 1).

The four response keys were "A", "S", "K", and "L" which were covered with stickers on a QWERTY keyboard, and the fingers participants’ used to press them were the Left/Right Middle/Index fingers—Lm, Li, Ri, and Rm, respectively (in the few novel-task-rules condition, the S-R association included only the index fingers).

Only stimuli mapped to the index fingers could appear during the NEXT phase. Thus, NEXT responses were either compatible or incompatible with the GO instructions. The following GO phase was indicated by the GREEN color of the stimuli. This GO phase consisted of two trials, in which two randomly chosen stimuli from those presented in the instructions appeared, thus requiring participants to be ready for all possible rules. At the end of each mini-block, a feedback regarding mean response times (RT) and number of errors in the GO phase appeared on the screen for 2 seconds. Each trial began with a 500 ms fixation point, followed by the target stimulus that appeared until participants responded, and ended with the presentation of a blank screen for 800 ms. Stimuli were (3x3 cm) Hebrew and English letters, digits, symbols, and pictures. Each mini-block included 2/4 stimuli from the same category (e.g., two pictures, four Hebrew letters).

#### Data analysis

The “prepdat” R package [31] was used for preprocessing. GO trials with an error were omitted from all RT analyses. Trials with a RT shorter than 100 ms (anticipation errors) or longer than 4,000 ms (outliers) were also omitted, as well as trials above three standard deviations for each participant, per condition. The analyses of the results included both Null-Hypothesis Testing (NHT) and Bayesian results (using JASP 0.8.1.2 [32] to estimate the relative odds of H1 and H0 given the data (assuming equal priors)). We report BF_10_, the relative odds of H1 compared to H0, which allows accepting H0 (if BF_10_ < 0.33). Thus, our statistical inference is based on the Bayesian results.

Next, we elaborate on the approach we took in analyzing the data. The order of analyses does not reflect the ordering of the mini-block (instructions→NEXT→GO). Instead, it follows the theoretical focus. We thus analyze GO performance first, because this is where we had the clearest predictions. We then continue with the NEXT phase, and then end with the analyses of instructions’ study times.

First, theoretically, first-trial performance is considered the purest measure of RITL, as it cannot rely on any previous task implementation [8]. In addition, empirical evidence shows that performance on the first GO trial is worse (slower and less accurate) than on the second GO trial; and that this difference was influenced by the degree of preparation towards the GO phase (see [5] Experiment 4). Therefore, in the following experiments, we first observe that this difference (termed “GO Trial effect”) is replicated. Only afterwards, we continue with the core analyses which examine whether performance in the first GO trial was influenced by the WM load manipulation.

Second, we similarly examined whether the NEXT compatibility effect was influenced by the WM load manipulation in the first and more advanced trials, separately. Specifically, there are indications that the NEXT effect observed in the first NEXT trial differs from that seen in subsequent trials. The indications include (a) the NEXT effect observed in the first NEXT trial tends to be numerically larger than in subsequent trials [5,24] although the related statistical interaction rarely reaches significance (see [5] Experiment 3, for an exception). This tendency is in line with other theories suggesting that participants learn to ignore irrelevant information with practice [33]; (b) the first NEXT trial is also characterized by general slowing relative to subsequent NEXT trials [34,35]. These considerations suggest that the NEXT effect in the first trial might serve as an optimal condition for NEXT effects to be seen (see S1 Appendix). Therefore, in the following experiments, we examined whether the NEXT compatibility effect is influenced by the load manipulation, and did so separately for the first NEXT trial and for the more advanced NEXT trials.

### Results and discussion

#### GO phase

RT and error rates were analyzed in a two-way ANOVA/BANOVA with the within-subjects independent variables Number of Task-Rules (2 vs. 4 novel task-rules) and GO Trial (Trial 1 vs. Trial 2). The results are pooled across mini-blocks involving at least one NEXT trial (i.e., 90% of the mini-blocks; since GO performance after zero NEXT trials was found to be weakly related to later GO performance [18]). Finally and very importantly, the results are reported for GO trials involving responses with the index fingers. This precaution was taken to strictly equate the conditions in terms of stimulus familiarity (only index-finger-related stimuli were presented in the NEXT phase which preceded the GO phase that is being analyzed) and in terms of responding fingers (only index fingers were used in the few novel-task-rules condition).

**Fig 2:**
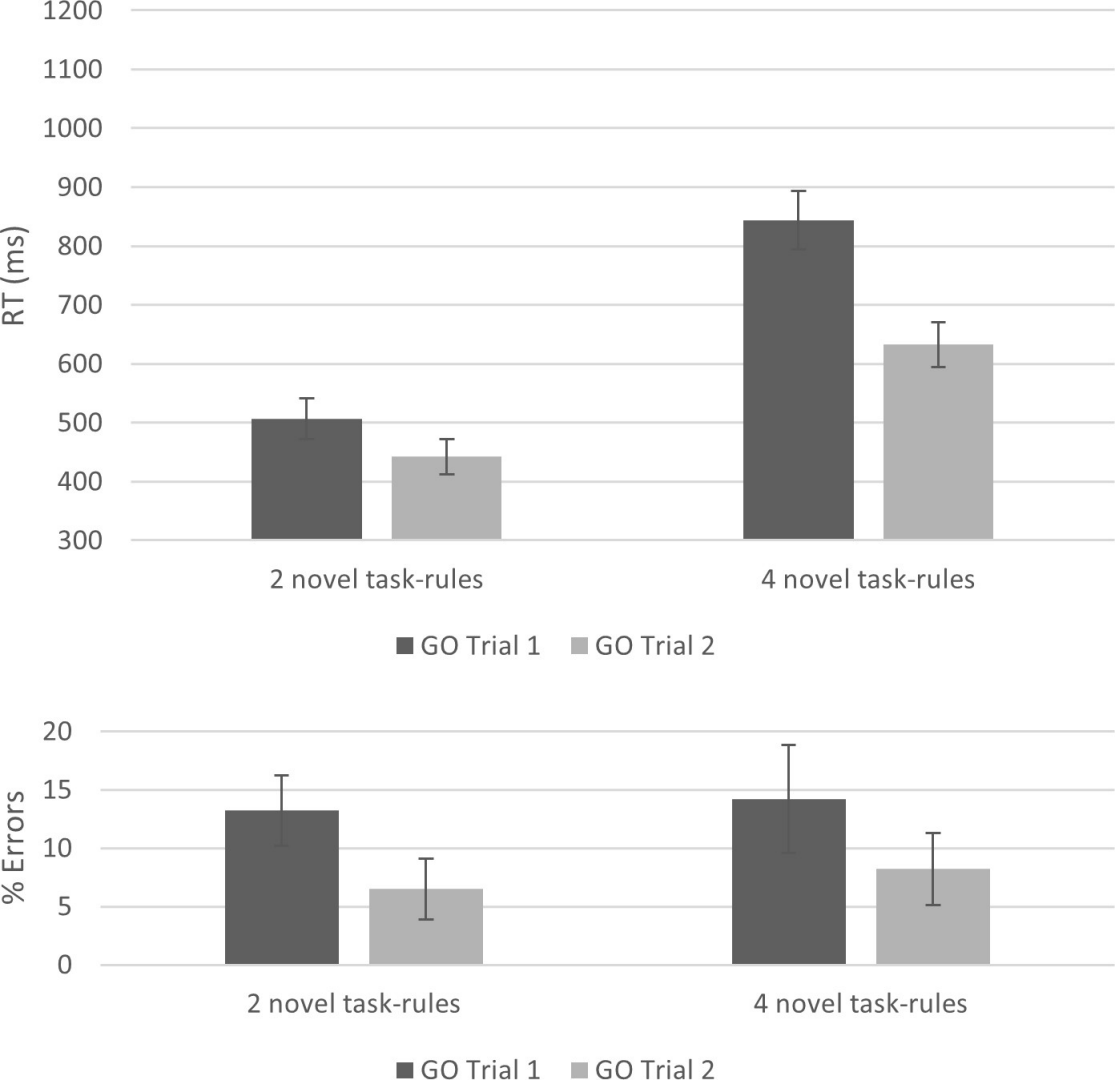
A two-way interaction between number of task-rules and GO trial in Experiment 1. The interaction demonstrates an increased RT GO Trial effect under high WM load (4 novel task-rules condition) (RT upper panel and error rates lower panel). Error bars represent Bayesian 95% Credible Intervals.

In RT, both main effects were robust. The Number of Task-Rules main effect [*F*(1, 21) = 134.70, *p* < .001, Ƞ^2^_p_ = .86, BF_10_ = 4.059e^+12^] shows significantly longer RT in the 4 novel task-rules condition relative to the 2 novel task-rules condition (738 ms vs. 474 ms, respectively). As in previous experiments, the results also indicated a robust main effect for GO Trial, supporting slower performance in the first relative to the second GO trial [*F*(1, 21) = 60.92, p < .001, Ƞ^2^_p_ = .74, BF_10_ = 82.82]. Finally, the interaction between Number of Task-Rules and GO Trial was robust, as well [*F*(1, 21) = 32.36, *p* < .001, Ƞ^2^_p_ = .61, BF_10_ = 126.16] (Fig 2, upper panel), showing a larger RT GO Trial effect in the 4 novel-task-rules condition. In error rates, only the main effect for GO Trial was robust [*F*(1, 21) = 14.53, *p* = .001, Ƞ^2^_p_ = .41, BF_10_ = 227.78]. Both the main effect for Number of Task-Rules and the interaction between Number of Task-Rules and GO Trial showed a null effect (*F*s < 1, BF_10_ < 0.30), suggesting that WM load significantly influenced only RT. Nonetheless, numerically, the mean error rate was 9.85% in the 2 novel-task-rules condition and 11.2% in the 4 novel-task-rules condition, suggesting that the results cannot be explained by speed-accuracy tradeoff.

#### NEXT phase

Since the NEXT phase did not involve a choice, NEXT errors were not monitored in this experiment, and thus only RTs were analyzed. Due to the special status of the first NEXT trial discussed earlier, the following analyses examine the first NEXT trial and more advanced NEXT trials separately.

**Fig 3:**
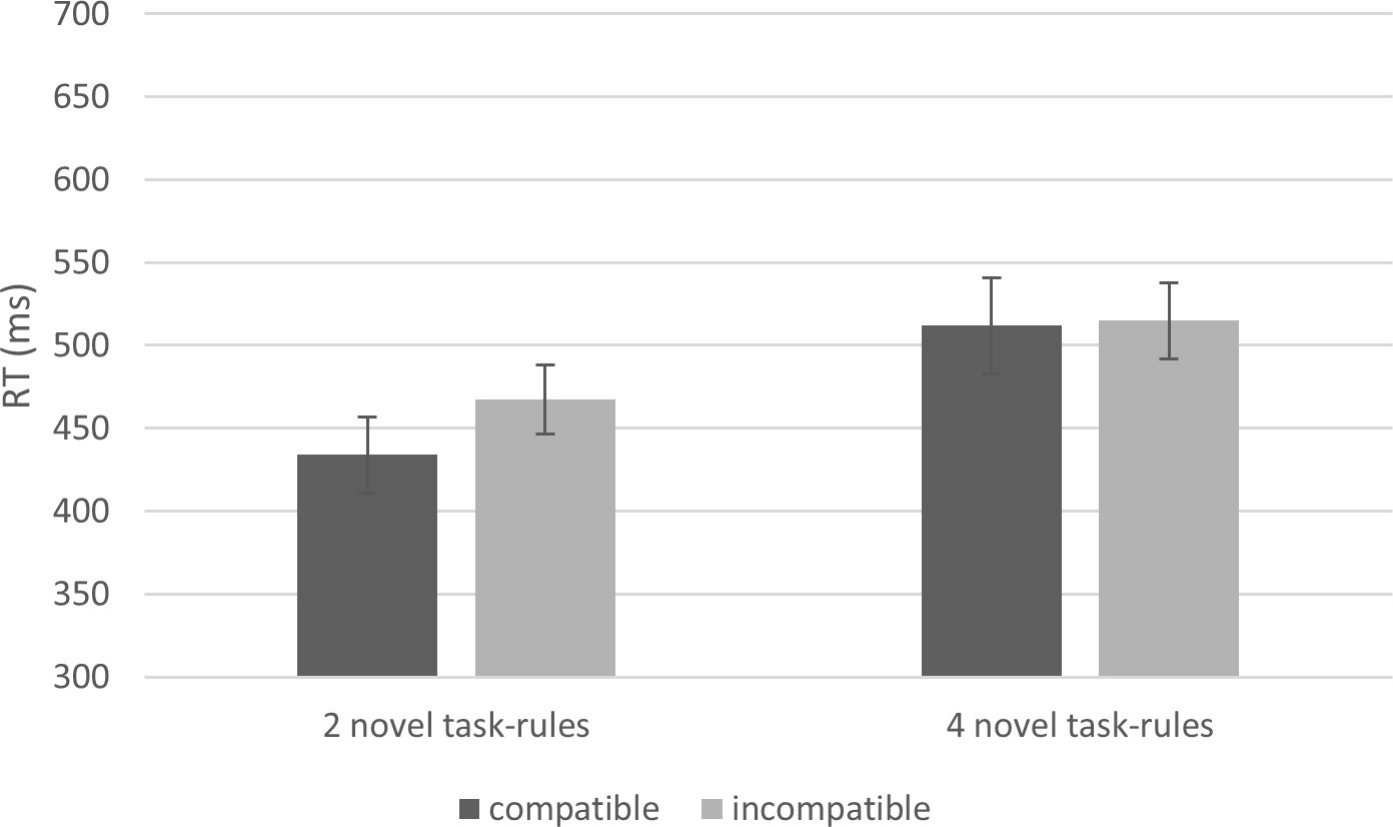
First NEXT trial RTs in Experiment 1. The two-way interaction between Number of Task-Rules and Compatibility showed anecdotal support for H0. Error bars represent Bayesian 95% Credible Intervals.

In the first NEXT trial, we examined whether the NEXT compatibility effect was influenced by the load manipulation using ANOVA and BANOVA. This analysis demonstrated a robust main effect for Number of Task-Rules [*F*(1,21) = 21.96, *p* < .001, Ƞ^2^_p_ = .51, BF_10_ = 12,084.91], a significant (by NHT) yet indecisive (by Bayesian inference) main Compatibility effect [*F*(1,21) = 2.80, *p* = .11, Ƞ^2^_p_ = .12, BF_10_ = 0.46]. Most importantly, the results showed an indecisive interaction between Compatibility and Number of Task-Rules [*F*(1, 21) = 2.33, *p* = .14, Ƞ^2^_p_ = .10, BF_10_ = 0.61], considered anecdotal evidence towards H0 (numerically, the NEXT compatibility effect was reduced from 33 ms in the 2 novel-task-rules condition, to 3 ms in the 4 novel-task-rules condition; see Fig 3).

In the more advanced NEXT trials (pooled), the results were similar, with a robust Number of Task-Rules main effect [*F*(1,21) = 22.17, *p* < .001, Ƞ^2^_p_ = .51, BF_10_ = 200,395.73], but a robust lack of Compatibility main effect [*F*(1,21) = 1.11, *p* = .30, Ƞ^2^_p_ = .05, BF_10_ = 0.28], and lack-of interaction between Number of Task-Rules and Compatibility, that supported H0 [*F*(1,21) = 0.12, *p* = .73, Ƞ^2^_p_ < .01, BF_10_ = 0.31, meaning that BF_01_ = 1.00/0.31 = 3.23]. Numerically, the NEXT compatibility effect was reduced from 9 ms to 5 ms by the WM load manipulation.

#### Study times

We recorded study times of the instructions screen towards the GO phase. These times include the 3s delay we forced on this screen. This analysis serves as a sanity check, assuming that it would take longer to study 4 novel task-rules, relative to 2 novel task-rules. As expected, participants took longer on average to encode the instructions in the high WM load condition involving 4 novel-task-rules (19 seconds) relative to the low WM load condition involving 2 novel-task-rules (6 seconds); a difference that was found to be significant (in NHT) in a one-sided t-test [*t*(21) = 6.10, *p* < .001, Cohen’s d = 1.30] and as providing very strong evidence supporting H1 (according to Jeffreys [36]), BF_10_ = 8,632.00.

To summarize, adding novel S-R mapping rules increased GO RT (but not errors), but did not substantially influence the NEXT compatibility effect (although it did influence NEXT general RT). However, the WM load manipulation we used was confounded with the probability that a prepared rule would be used in the GO phase (higher with 2 than with 4 task-rules). Importantly, the likelihood of future usage has previously been shown to influence automatic effects of instructions [37]. Thus, it is conceivable that the numeric reduction in the NEXT compatibility effect in the 4 novel task-rules condition (assumed to involve high WM load) reflects the reduction in expected future usage and not load. Therefore, in Experiment 2, we wanted to test whether an increase in the number of task-rules (with associated reduction in expectancy of future usage) would influence the NEXT compatibility effect, even if the increase in number of rules comes with minimal, if any, involvement of WM.

## Experiment 2

Experiment 2 involved two groups of participants. In each group, we employed a within-subjects manipulation of Number of Task-Rules, and the groups differed in whether an increase in Number of Task-Rules was associated with an increase in WM load. In one group, (novel-rules-load group) we replicated Experiment 1. This group accordingly involved two conditions–low WM-load with 2 novel task-rules (lWM2R), and high WM load with 4 novel task-rules (hWM4R). In the second group of participants (familiar-rules-load group), the 2 novel task-rules condition was the same as in the novel-rules-load group, but the 4-task-rules condition was different. It involved 2 novel task-rules + 2 *familiar* task-rules that are thus not assumed to rely on WM (lWM4R, meaning *low*-WM with 4 task-rules). Our prediction was that, if the critical variable is the number of Task-Rules and *not* WM load, the effects seen in Experiment 1 would replicate in both groups. If, however, the critical variable is WM load, then the effects of Experiment 1 would replicate only in the novel-rules-load group and would be absent or substantially reduced in the familiar-rules-load group.

### Method

#### Participants

Given that in Experiment 1 we did *not* observe a significant influence of the WM load manipulation on the NEXT compatibility effect, we sought to increase the statistical power and determined a sample size that would be sufficient to detect a smaller effect size of Ƞ^2^_p_ = 0.045 (Power = .80) in a mixed between-within design. Accordingly, a new sample comprising seventy-one Ben-Gurion University of the Negev undergraduate students (59 women, *M*_age_ = 23.06, *SD* = 1.24), with similar attributes to participants from Experiment 1, took part in the experiment in return for course credit. Participants were randomly assigned to one of the two groups.

(Technical note: One participant from the novel-rules-load group was removed from the analyses after leaving mid-experiment, since she failed to finish the experiment within the estimated time, due to extremely long times to study the rules. Whereas other participants in that group took between 4 to 14 seconds in the 2 novel task-rules condition, and between 4 to 32 seconds in the 4 novel task-rules condition, this participant took 15 and 52 seconds to study the instructions in the two conditions, respectively).

#### Materials and procedure

The procedure in the novel-rules-load group was identical to Experiment 1. The novel-rules-load group received identical conditions to those of Experiment 1, except for the marking of eligible response alternatives (described below). In the familiar-rules-load group, the 2 novel task-rules condition (lWM2R) was identical to that of the novel-rules-load group. The 2 novel + 2 familiar task-rules condition (lWM4R) included two novel task-rules (related to the index fingers) and two familiar task-rules (arrows, related to the middle fingers). In each group, there were 35 mini-blocks with four task-rules, and 35 mini-blocks with two task-rules. The rules associated with the left and right index fingers were always novel. In the 4 task-rules conditions, the added rules (associated with the left and right middle fingers) were novel rules in the novel-rules-load group, and familiar rules in the familiar-rules-load group. The two familiar stimuli were left/right pointing arrows, which were mapped to the corresponding left/right middle finger.

The rules associated with the middle fingers served as a load task and (especially when arrows were used), participants could have conceived of the middle-fingers rules as belonging to another task. This could have confounded the critical manipulation—which is whether the additional alternatives add WM load; and could instead be perceived as—whether the four task-rules belong to one task or two tasks. To prevent this confound, we induced the 2-task strategy in both groups, making them similar in this respect. Specifically, we followed Shahar et al. (see [16], Experiment 3) and added four squares below the target-stimulus, representing the four response keys (2 on each side of the screen, see Fig 1 lower panel). In each trial, two asterisks appeared in either the two inner squares or the two outer squares, pointing to whether the current target stimulus belongs to the "inner" or "outer" task set, respectively. It was further explained that during the NEXT phase, the asterisks would always point at the inner keys, and that the use of these cues is encouraged. We nonetheless note that most of the participants reported ignoring the asterisks in the post-experimental debriefing.

Due to a technical error, eight of the participants in the familiar-rules-load group received five less mini-blocks in each of the two conditions, but preliminary analyses indicated that their results did not differ from the rest of their group.

### Results and discussion

#### GO phase

**Fig 4:**
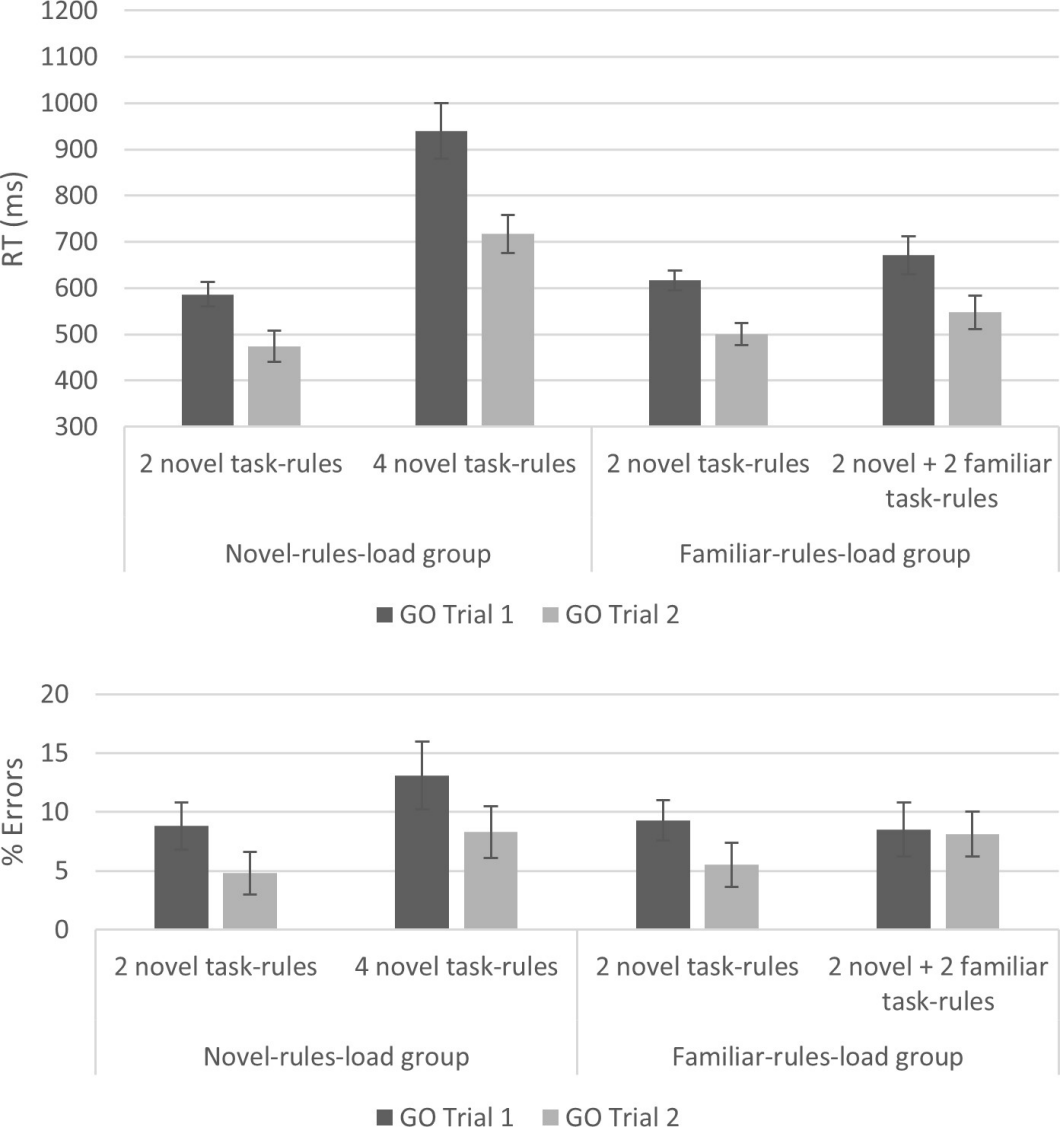
A three-way interaction between Group, Number of Task-Rules, and GO Trial in Experiment 2. The interaction demonstrates an increased RT GO trial effect under the 4 novel task-rules condition in the novel-rules-load group, but not in the 2 novel + 2 familiar task-rules condition in the familiar-rules-load group (RT upper panel and error rates lower panel). Error bars represent Bayesian 95% Credible Intervals.

We performed two 3-way ANOVAs and BANOVAs, with the between-subjects independent variable Group, and the within-subjects independent variables GO Trial (Trial 1 vs. Trial 2) and Number of Task-Rules (2 vs. 4), on RT and error rates. The results were again pooled across mini-blocks involving 1–5 NEXT trials, and only included those trials that required responding with the index fingers. In RT, the results indicated a main effect for GO Trial [*F*(1, 68) = 105.78, *p* < .001, Ƞ^2^_p_ = .61, BF_10_ = 2.79e^+8^], showing slower RT in the first vs. second GO Trial. In addition, there were main effects for Number of Task-Rules [*F*(1, 68) = 197.82, *p* < .001, Ƞ^2^_p_ = .74, BF_10_ = 10.4e+17], indicating slower RT in the 4- vs. 2-task-rules conditions; and Group [*F*(1, 68) = 9.85, *p* < .01, Ƞ^2^_p_ = .13, BF_10_ = 9.02], indicting slower RT in the novel-rules-load group relative to the familiar-rules-load group. Most importantly, the prediction of a three-way interaction between Group, Number of Task-Rules, and GO Trial was supported [*F*(1, 68) = 8.54, *p* < .01, Ƞ^2^_p_ = .11, BF_10_ = 4.55; Fig 4]. This triple interaction shows that the GO trial effect was only increased by novel mapping rules (lWM2R vs. hWM4R in the novel-rules-load group; *F*(1, 34) = 9.42, *p* < .01, Ƞ^2^_p_ = .22, BF_10_ = 4.51), but not by familiar mapping rules (lWM2R vs. lWM4R in the familiar-rules-load group; *F*(1, 34) = 0.55, *p* = .46, Ƞ^2^_p_ = .02, BF_10_ = .32). In error rates, there were main effects for GO Trial [*F*(1, 68) = 20.11, *p* < .001, Ƞ^2^_p_ = .23, BF_10_ = 397.39] and Number of Task-Rules [*F*(1, 68) = 8.10, *p* < .01, Ƞ^2^_p_ = .11, BF_10_ = 10.21], as in RT; but there was no main effect for Group [*F*(1, 68) = 0.62, *p* = .43, Ƞ^2^_p_ < .01, BF_10_ = 0.26], suggesting that errors rates were not generally increased in the novel-rules-load group. In addition, none of the interactions involving Number of Task-Rules and Group was robust (all *F*s < 3.5, BFs_10_ < 1.2). Importantly, there was no indication for speed-accuracy tradeoff (see Fig 4).

#### NEXT phase

As in Experiment 1, we tested the NEXT compatibility effect for the first and more advanced trials, separately; and in addition we did so for each group separately, as well.

We first performed two ANOVAs and BANOVAs (on the first and advanced, 2–5, NEXT trials, pooled), with the within-subjects variables Number of Task-Rules (2 vs. 4) and Compatibility (compatible vs. incompatible) in the novel-rules-load group. The results showed that participants were slower to respond in the 4 novel task-rules condition (hWM4R), in both the first and advanced NEXT trials [i.e., there was a robust main effect for Number of Task-Rules for both the first NEXT trial: *F*(1, 34) = 68.38, *p* < .001, Ƞ^2^_p_ = .67, BF_10_ = 9.120e^+7^; and advanced NEXT trials: *F*(1, 34) = 14.99, *p* < .01, Ƞ^2^_p_ = .31, BF_10_ = 8,593.68]. The results again indicate a significant (by NHT) yet indecisive (Bayesian) main effect for Compatibility in the first NEXT trial [*F*(1, 34) = 7.64, *p* < .01, Ƞ^2^_p_ = .18, BF_10_ = 2.37], but a robust effect in the advanced NEXT trials [*F*(1, 34) = 9.12, *p* < .01, Ƞ^2^_p_ = .21, BF_10_ = 3.56]. However, no modulation of the NEXT compatibility effect was supported by the results, which allowed us to accept the null hypothesis [for first NEXT trial, Number of Task-Rules *Compatibility interaction: *F*(1, 34) = 0.7, *p* = .41, Ƞ^2^_p_ = .02, BF_10_ = .33; for advanced NEXT trials, Number of Task-Rules *Compatibility interaction: *F*(1, 34) = 1.86, *p* = .18, Ƞ^2^_p_ = .05, BF_10_ = .29].

We repeated the same analyses in the familiar-rules-load group. Unlike in the novel-rules-load group, the Number of Task-Rules main effect was indecisive [for first NEXT trial: *F*(1, 34) = 17.42, *p* < .001, Ƞ^2^_p_ = .34, BF_10_ = 1.64; for advanced NEXT trials: *F*(1, 34) = 4.24, *p* < .05, Ƞ^2^_p_ = .11, BF_10_ = .91]; and the NEXT Compatibility effect was robust in all NEXT trials [for first NEXT trial: *F*(1, 34) = 33.95, *p* < .001, Ƞ^2^_p_ = .50, BF_10_ = 3.402e^+8^; for advanced NEXT trials: *F*(1, 34) = 17.03, *p* < .001, Ƞ^2^_p_ = .33, BF_10_ = 1,951.66]. Most importantly, the NEXT compatibility effect was, again, uninfluenced by the increased number of task-rules [for first NEXT trial: *F*(1, 34) = 1.02, *p* = .32, Ƞ^2^_p_ = .03, BF_10_ = .33; for advanced NEXT trials: *F*(1, 34) = 0.87, *p* = .36, Ƞ^2^_p_ = .02, BF_10_ = .31], allowing us to accept H0. The results thus far suggest that while the NEXT compatibility effect was uninfluenced by an increase in the number of task-rules (whether WM demanding or not), the NEXT general RT was only influenced by the Number of Task-Rules when it was also associated with a WM load increase.

**Fig 5:**
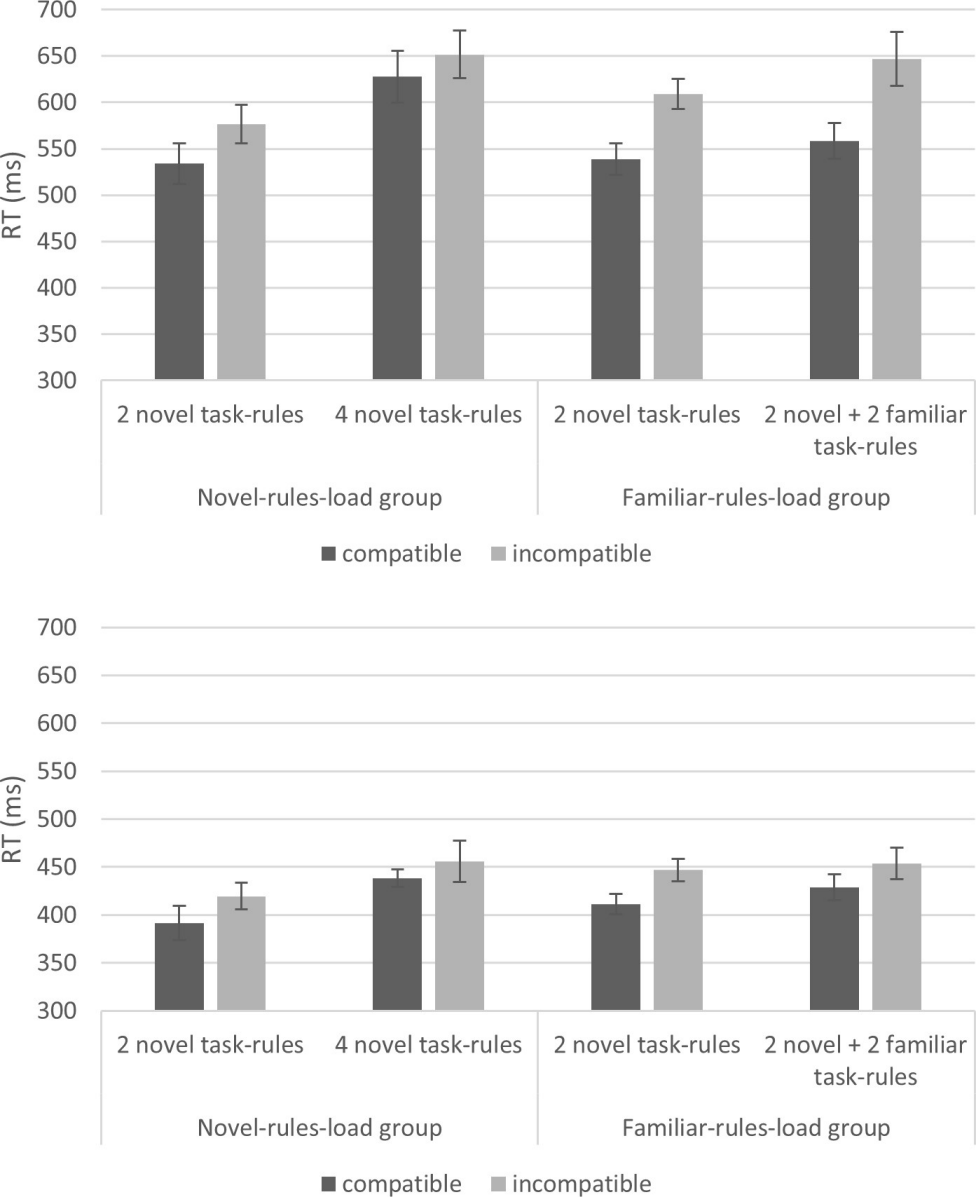
**NEXT RT in Experiment 2 for the first NEXT trial (upper panel) and advanced NEXT trials (lower panel).** The results did not show a robust influence of Number of Task-Rules on the NEXT compatibility effect. Error bars represent Bayesian 95% Credible Intervals.

Finally, in order to directly compare the groups (see Fig 5), we entered both groups into a single ANOVA and BANOVA, with the within-subjects independent variables Number of Task-Rules and Compatibility, and the between-subjects independent variable Group. These analyses were conducted on the first and advanced NEXT trials, separately.

For the first NEXT trial, the results showed a robust two-way interaction between Number of Alternatives and Group [*F*(1, 68) = 20.61, *p* < .001, Ƞ^2^_p_ = .23, BF_10_ = 20.73], demonstrating that only participants in the novel-rules-load group were slowed in their first NEXT RT by the increase in the number of task-rules. However, the three-way interaction between Group, Number of Task-Rules, and Compatibility was indecisive [*F*(1, 68) = 1.65, *p* = .20, Ƞ^2^_p_ = .02, BF_10_ = .49], with size considered as anecdotal evidence towards H0. In the advanced NEXT trials, a similar pattern was observed, only that the three-way interaction allowed the acceptance of H0 [two-way Number of Task-Rules *Group interaction: *F*(1, 68) = 5.63, *p* = .02, Ƞ^2^_p_ = .08, BF_10_ = 4.97; three-way Number of Task-Rules *Group*Compatibility interaction: *F*(1, 68) < 0.1, *p* = .99, Ƞ^2^_p_ < .01, BF_10_ = .32].

#### Study times

Similar to Experiment 1, we wanted to make sure that participants took longer to learn 4 task-rules relative to 2 task-rules, especially when the rules were novel (novel-rules-load group). The results support the expected pattern, showing a relatively small increase in study times with increasing number of task-rules from 5.8 to 6.5 sec in the familiar-rules-load group. In contrast, in the novel-rules-load group, the effect was considerably larger, as it took 5.2 vs. 10.5 sec on average to encode the instructions in the 2 vs. 4 novel task-rules conditions, respectively. We analyzed these results in an ANOVA and BANOVA with Number of Task-Rules (2 vs. 4) as the within-subjects independent variable, and Group as the between-subjects independent variable, and the results indeed supported a robust interaction [*F*(1, 68) = 32.82, *p* < .001, Ƞ^2^_p_ = .33, BF_10_ = 44,999.99].

Experiment 2 was run in order to test an alternative account that the critical variable is Number of Task-Rules and not WM load. The results clearly rule out this account since the results of Experiment 1 were replicated only when the increase in the number of task-rules was associated with a WM load increase (i.e., in the novel-rules-load group). As in Experiment 1, the NEXT compatibility effect was not influenced by these manipulations, but NEXT general RT was influenced in a similar manner to GO RT.

Nonetheless, another alternative account remains. In both the 2 novel task-rules condition and in the 2 novel + 2 familiar task-rules condition in the familiar-rules-load group, the response options could be easily categorized as “left” and “right”. This is obvious when there were 2 task-rules, but also when there were four task-rules: the two novel rules could be labeled as “right” and “left” and the other two (familiar) rules were associated with arrows, which were also easily labeled as “right” and “left”. In contrast, a similar categorization was less possible when there were 4 novel task-rules in the novel-rules-load group, as these rules could not be easily categorized to two group. This analysis suggests that the results might actually reflect differences in response coding rather than load proper.

To address this issue, in the third experiment, we used a procedure in which the two non-load responses (associated with the index fingers) could be categorized as left/right, whereas the additional (load) rules were not associated with right/left responses, but instead involved a simultaneous key press by the two equivalent fingers of *both* hands (e.g., simultaneous pressing of the two middle fingers).

Another modification introduced in Experiment 3 involved enhancement of the load manipulation. Following Ellenbogen and Meiran [38], who demonstrated that loading WM with 4 novel rules was insufficiently powerful, we increased the load from 4 to 6 task-rules. We reasoned that such a high load might result in a modulation of not only GO performance, but also the NEXT compatibility effect. That is, it is possible that the NEXT and GO phases have differential sensitivity to WM manipulations, suggesting that an extreme load condition has the potential to reveal WM load sensitivity, even differentially for NEXT phase compatibility responses conditions.

## Experiment 3

The 2 novel task-rules conditions in both groups were similar to that used in the previous experiments, except that the left and right response keys were placed closer to each other. The 6 task-rules conditions included 4 additional rules involving responses that had to be simultaneously performed with both hands, such that they could not be represented as right/left (see Method for an elaboration). In the novel-rules-load group, these 4 additional rules were all novel and arbitrary, as in the previous experiments. In the familiar-rules-load group, these additional rules comprised of S-R mappings that were non-arbitrary. Specifically, the stimuli were designed in such a way that they would clearly remind what the response should be.

### Method

#### Participants

A new sample of forty-six Ben-Gurion University of the Negev undergraduate students (41 women, *M*_age_ = 23.06, *SD* = 0.99), with similar attributes to participants from the previous experiments, took part in the experiment in return for course credit. Participants were randomly assigned to the novel- and familiar-rules-load groups. The sample size was determined as double the size of Experiment 1 (since it involves a between-subjects factor).

#### Materials and procedure

**Fig 6:**
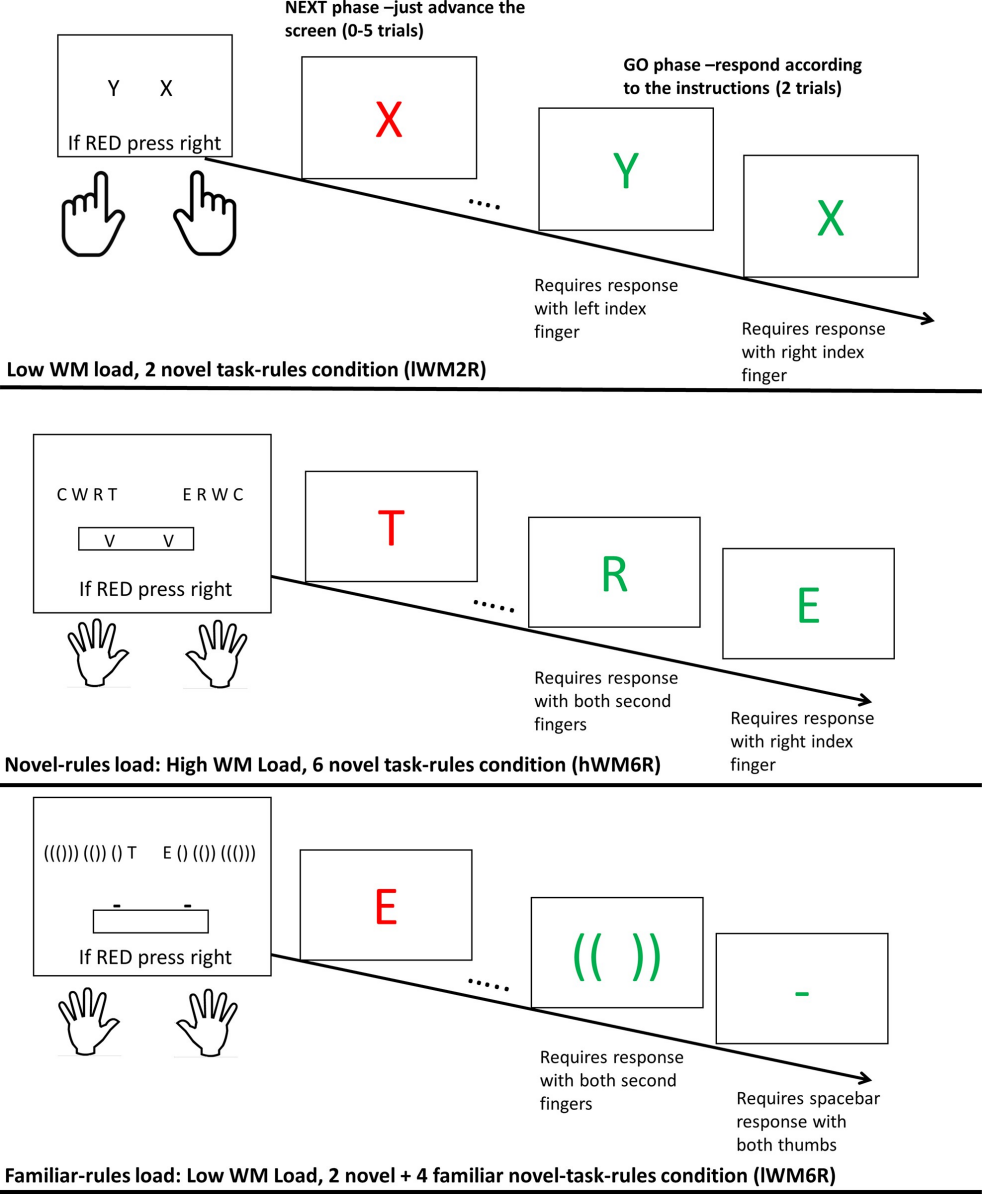
Mini-block structure in Experiment 3. Upper panel: both groups’ lWM2R condition involving 2 novel task-rules; middle panel: novel-rules-load group hWM6R condition involving 6 novel task-rules; lower panel: familiar-rules-load group lWM6R condition involving 2 novel task-rules + 4 familiar task-rules. Notice that in the 6 task-rules conditions, the additional rules which required simultaneous responses with both hands appeared twice on the instruction screen to demonstrate that both hands are required.

The 2 novel task-rules condition in both groups was similar to that of Experiments 1 and 2 and included two novel rules. The only difference to the previous experiments was that the two response keys were “F” and “H” on a QWERTY keyboard. Since these keys are fairly close to each other, the stimuli on the instructions screen were presented closer together, as well (see Fig 6). The 6 task-rules conditions included six S-R mapping rules in total. In addition to two novel rules (associated with “right” and “left” responses), four additional rules were added. These rules associated stimuli with responses that had to be executed simultaneously using both hands, with corresponding left and right fingers. They were associated with the simultaneous pressing of left + right middle fingers (“D+J”), left + right ring fingers (“S+K”), left + right pinky fingers (“A+L”), and left + right thumbs (on the spacebar). Given that these simultaneous responses were relatively difficult to execute, we gave participants a relatively long practice involving 20 trials that was meant to make sure that they were able to execute these responses correctly. For these responses, the program was set to accept only responses in which the participant used both hands, as required. For example, if the required response was “S+K”, but the participant pressed “S”, this was considered an error. At the end of each mini-block, participants received feedback regarding their performance during the GO phase, and they were encouraged to try to continuously improve their performance (be as quick and as accurate as possible) throughout the experiment. Additionally, in this version of the task, we collected NEXT errors (see [22]), though participants still had to (eventually) press the NEXT response in order to advance to the GO phase. Finally, since participants tended to ignore the asterisks in Experiment 2, we dropped these cues in the current experiment.

The stimuli in the novel-rules-load group were the same as in Experiments 1 and 2. In the familiar-rules-load group, the additional stimuli were designed to induce the appropriate response (i.e., to be non-arbitrary). They were first presented during the general instruction phase and were also explained orally to the participants. The stimuli requiring responses by the middle fingers, ring fingers, and pinky fingers were constructed as a number of parentheses corresponding to the responding finger (for example, “((()))” to signal the middle finger; see Fig 6). For the thumbs, the stimulus was a long horizontal bar, similar to the spacebar. During the instructions of the 6 task-rules conditions (in both groups), the stimuli which required simultaneous responses with both hands appeared twice on the screen, in locations mirroring their appropriate responses (Fig 6, middle and lower panels), in order to facilitate the simultaneous nature of the responses.

### Results

#### GO phase

**Fig 7:**
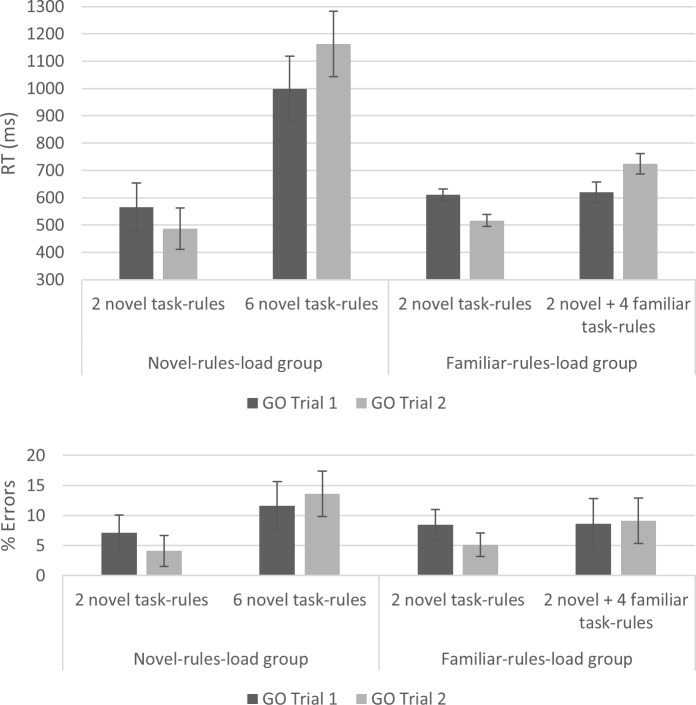
A three-way interaction between Group, Number of Task-Rules, and GO Trial in Experiment 3. The interaction demonstrates that Number of Task-Rules influenced RT in both groups (upper panel), but only in the novel-rules-load group it also influenced error rates (lower panel). Error bars represent Bayesian 95% Credible Intervals.

As in Experiment 2, RT and errors (only for trials requiring responses with the index fingers) were analyzed in a three-way ANOVA and BANOVA with the between-subjects variable Group, and the within-subjects variables Number of Task-Rules (2 vs. 6) and GO Trial (1 vs. 2). To our surprise, the results indicated an unusual finding: The main effect for GO Trial was absent in both RT and in errors [RT: *F*(1, 46) = 2.38, *p* = .13, Ƞ^2^_p_ = .05, BF_10_ = 0.18; error rates: *F*(1, 46) = 0.79, *p* = .38, Ƞ^2^_p_ = .02, BF_10_ = .22]. In addition, GO Trial interacted with Number of Task-Rules in RT [*F*(1, 46) = 37.80, *p* < .001, Ƞ^2^_p_ = .45, BF_10_ = 63.44], albeit not in errors [*F*(1, 46) = 4.26, *p* < .05, Ƞ^2^_p_ = .08, BF_10_ = 1.37]. This interaction in RT shows a general reversal of the GO trial effect in the 6 task-rules condition with a similar non-significant trend in errors, and without a difference between the groups [*F*(1, 46) = 0.38, *p* = .54, Ƞ^2^_p_ < .01, BF_10_ = 0.32]. Given that this pattern was seen *in both groups* (Fig 7), it suggests that either motor complexity or the high number of task-rules, and not the WM-related aspects of the hWM6R condition, caused this reversal. As noted in the Introduction, this suggests that the GO trial effect no longer has its original implication in this experiment, and in the following analyses, we focus on the first GO trial, which, as was previously noted, is the clearest indication of RITL.

Therefore, we entered the between-subjects independent variable Group and the within-subjects independent variable Number of Task-Rules to an ANOVA and BANOVA. In RT, the results indicated a robust interaction between Number of Task-Rules and Group [*F*(1, 46) = 26.32, *p* < .001, Ƞ^2^_p_ = .36, BF_10_ = 13,284.50], demonstrating greater slowing with Number of Task-Rules increase in the novel-rules-load group (from 565 ms to 999 ms), relative to the familiar-rules-load group (from 610 ms to 620 ms). Probing this interaction showed a robust effect in the novel-rules-load group [*F*(1, 23) = 28.76, *p* < .001, Ƞ^2^_p_ = .56, BF_10_ = 18,881.62], but a null effect in the familiar-rules-load group [*F*(1, 23) = 0.29, *p* = .59, Ƞ^2^_p_ = .01, BF_10_ = 0.32]. In errors, the interaction between Group and Number of Task-Rules was indecisive [*F*(1, 46) = 1.73, *p* = .19, Ƞ^2^_p_ = .04, BF_10_ = 0.71], but the descriptive pattern was in the same direction as RT (error rates increased with load from 7.1% to 11.6% in the novel-rules-load group; and from 8.5% to 8.6% in the familiar-rules-load group).

Although the results focused on the first GO trial, they are similar to those of previous experiments, showing that GO RT were robustly influenced by a novel-rules-load and not by familiar-rules-load.

#### NEXT phase

NEXT RT and error rates were analyzed for the first and more advanced trials, separately, as in the previous experiments. The results were generally similar to those of Experiment 2. We first examined the novel-rules-load group. We performed two ANOVAs and BANOVAs (for the first and advanced trials, separately), with the within-subjects independent variables Number of Task-Rules and Compatibility. The results show a robust effect for Compatibility [for the first NEXT trial: *F*(1, 23) = 18.84, *p* < .001, Ƞ^2^_p_ = .45, BF_10_ = 9.91; for the advanced NEXT trials: *F*(1, 23) = 17.97, *p* < .001, Ƞ^2^_p_ = .44, BF_10_ = 6.84]. In addition, there was a robust effect for Number of Task-Rules [for the first NEXT trial: *F*(1, 23) = 41.79, *p* < .001, Ƞ^2^_p_ = .64, BF_10_ = 1.34e^+7^; for the advanced NEXT trials: *F*(1, 23) = 60.98, *p* < .001, Ƞ^2^_p_ = .73, BF_10_ = 2.59e^+9^]. However, the NEXT compatibility effect was not robustly influenced by this general increase in RT [for the first NEXT trial, considered anecdotal evidence towards H0: *F*(1, 23) = 2.21, *p* = .15, Ƞ^2^_p_ = .09, BF_10_ = 0.46; for the advanced NEXT trials, considered strong evidence for H0: *F*(1, 23) = 0.63, *p* = .43, Ƞ^2^_p_ = .03, BF_10_ = 0.28].

The same set of analyses was performed on NEXT error rates in this group (novel-rules-load). The ANOVA and BANOVA on the first NEXT trial showed a robust main effect for Compatibility [*F*(1, 23) = 19.43, *p* < .001, Ƞ^2^_p_ = .46, BF_10_ = 102.53]. A robust main effect was found for Number of Task-Rules as well [*F*(1, 23) = 4.68, *p* = .04, Ƞ^2^_p_ = .17, BF_10_ = 3.50], but again there was no modulation of the NEXT compatibility effect by Number of Task-Rules [*F*(1, 23) = 1.62, *p* = .22, Ƞ^2^_p_ = .07, BF_10_ = 0.56], with an indecisive result considered as anecdotal evidence towards H0 (increase from a compatibility effect of 3% to 5.6% errors). On advanced NEXT trials, the main effect for Number of Task-Rules was indecisive and showed anecdotal evidence towards H0 [F(1,23) = 2.61, p = .12, Ƞ^2^_p_ = .10, BF_10_ = 0.56]. The main Compatibility effect was indecisive [*F*(1, 23) = 2.86, *p* = .10, Ƞ^2^_p_ = .11, BF_10_ = 1.21], and a similar result was obtained for the interaction between Number of Task-Rules and Compatibility [F(1,23) = 0.61, p = .44, Ƞ^2^_p_ = .03, BF_10_ = 0.38].

In the familiar-rules-load group, unlike in the novel-rules-load group, there was no main effect for Number of Task-Rules on NEXT RT [first NEXT trial: *F*(1, 23) = 1.07, *p* = .31, Ƞ^2^_p_ = .04, BF_10_ = 0.25; advanced NEXT trials: *F*(1, 23) = 0.91, *p* = .35, Ƞ^2^_p_ = .04, BF_10_ = 0.27]. While the main Compatibility effect was robust [first NEXT trial: *F*(1, 23) = 14.23, *p* < .001, Ƞ^2^_p_ = .38, BF_10_ = 23,165.08; advanced NEXT trials: *F*(1, 23) = 19.49, *p* < .001, Ƞ^2^_p_ = .46, BF_10_ = 23,280.79], it was not robustly influenced by the Number of Task-Rules [first trial: *F*(1, 23) = 1.05, *p* = .32, Ƞ^2^_p_ = .04, BF_10_ = 0.28; advanced trials: *F*(1, 23) = 1.39, *p* = .25, Ƞ^2^_p_ = .06, BF_10_ = 0.40].

The same pattern was seen in NEXT error rates, as well. There was a main effect for Compatibility [first trial: *F*(1, 23) = 19.08, *p* < .001, Ƞ^2^_p_ = .45, BF_10_ = 448,390.00; advanced trials: *F*(1, 23) = 12.43, *p* < .01, Ƞ^2^_p_ = .35, BF_10_ = 2,128.11], but no main effect for Number of Task-Rules [*F*s < 1, BFs_10_< .22], and the NEXT effect was not modulated by the Number of Task-Rules manipulation [*F*s < 1, BFs_10_ < .33].

Finally, for the sake of completeness, we performed a set of ANOVAs and BANOVAs to directly test the differences between the groups. Like in Experiment 2, these analyses included the between-subjects variable Group and the within-subjects variables Number of Task-Rules and Compatibility. First, in RT for the first NEXT trial, the results showed a robust Group* Number of Task-Rules interaction [*F*(1, 46) = 27.76, *p* < .001, Ƞ^2^_p_ = .38, BF_10_ = 1,450.41], and supported the lack of a three-way interaction between Group, Number of Task-Rules, and Compatibility [*F*(1, 46) = 0.18, *p* = .67, Ƞ^2^_p_ < .01, BF_10_ = 0.30]. The same pattern was observed for the advanced NEXT trials [Group* Number of Task-Rules interaction: *F*(1, 46) = 41.08, *p* < .001, Ƞ^2^_p_ = .47, BF_10_ = 1.36e+6; three-way interaction: *F*(1, 46) = 1.86, *p* = .18, Ƞ^2^_p_ = .04, BF_10_ = 0.48].

**Fig 8:**
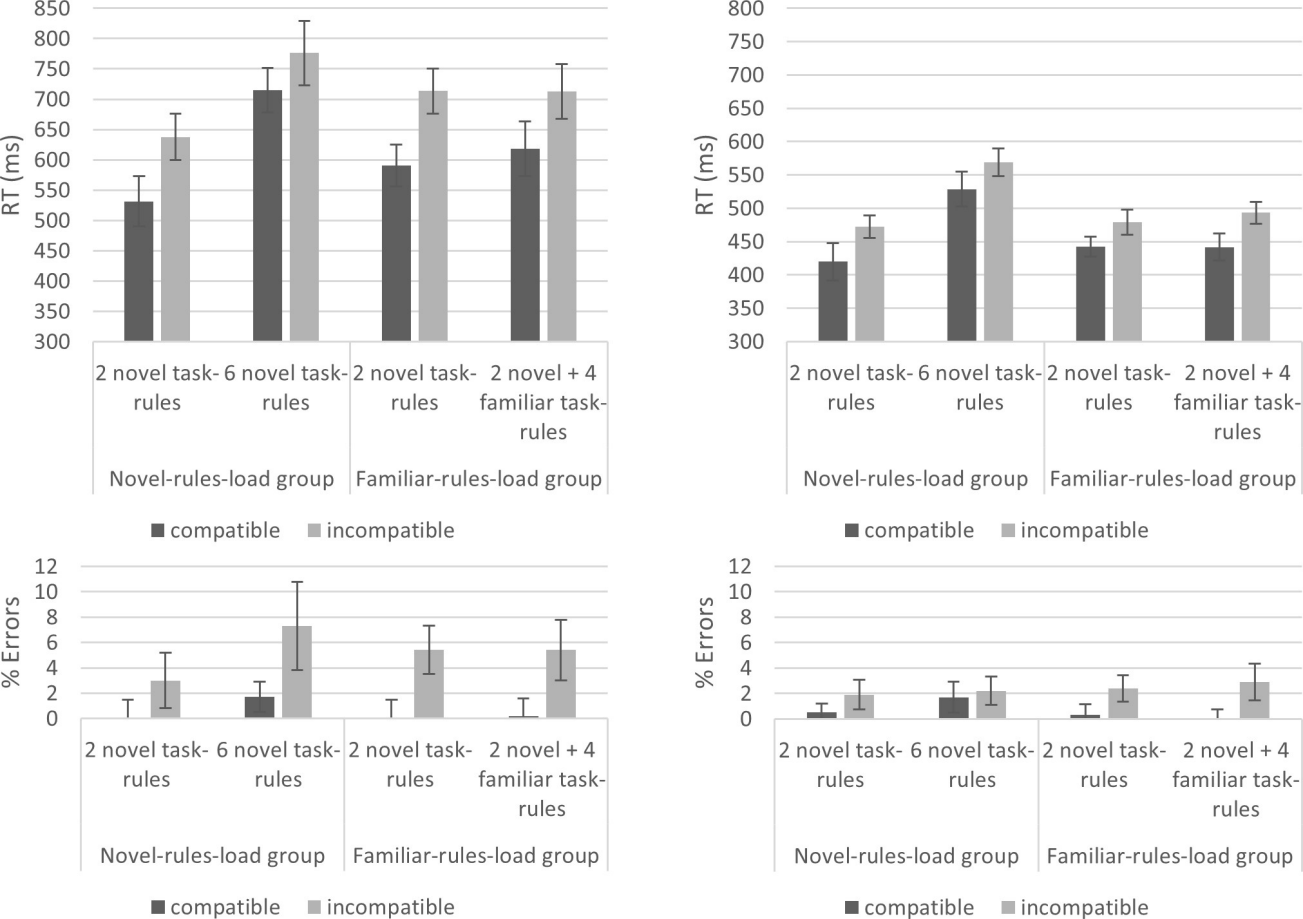
**NEXT RT (upper panels) and error rates (lower panels) in Experiment 3 for the first NEXT trial (left panels) and advanced NEXT trials (right panels).** The results did not show a robust influence of WM load on the NEXT compatibility effect. Error bars represent Bayesian 95% Credible Intervals.

In NEXT error rates, the results were similar, though without the indication of a robust two-way interaction between Number of Task-Rules and Group [first trial: *F*(1, 46) = 3.46, *p* = .07, Ƞ^2^_p_ = .07, BF_10_ = 1.07; advanced trials: *F*(1, 46) = 1.04, *p* = .31, Ƞ^2^_p_ = .02, BF_10_ = 0.29]. Similar to RT, the results concerning the three-way interaction between Group, Number of Task-Rules, and Compatibility indicated anecdotal evidence towards accepting H0 [first trial: *F*(1, 46) = 1.37, *p* = .25, Ƞ^2^_p_ = .03, BF_10_ = 0.44; advanced trials: *F*(1, 46) = 1.37, *p* = .25, Ƞ^2^_p_ = .03, BF_10_ = 0.60]. Taken together, similar to Experiment 2 the results suggest that, while only the novel-rules-load group was *generally* influenced by the Number of Task-Rules manipulation during the NEXT phase (and only in RT), Number of Task-Rules did not affect the difference between the compatible and incompatible conditions (Fig 8).

#### Study times

The average study time in the novel-rules-load group was 5.3 seconds in the lWM2R condition and 25.0 seconds in the hWM6R condition; whereas in the familiar-rules-load group, the average study time was 6.0 seconds in the lWM2R condition and 6.8 seconds in the lWM6R condition. The study times were entered into an ANOVA and BANOVA with the within-subjects variable Number of Task-Rules (2 vs. 6) and the between-subjects variable Group. The results resembled those of Experiment 2: They showed a very strong interaction between Number of Task-Rules and Group [*F*(1, 46) = 100.10, *p* < .001, Ƞ^2^_p_ = .68, BF_10_ = 9.56e^+11^]. This finding is interesting on its own, as it suggests that the manipulation worked, and participants did not need the extra time to encode the elaborate additional rules in the familiar-rules-load group.

In summary, the results generally replicated those of the previous experiments, demonstrating that first GO trial performance was influenced by novel-rules-load, but that the NEXT compatibility effect was uninfluenced by the manipulation (although a non-specific influence on NEXT RT was observed under novel-rules-load). The contribution of the present experiment was in ruling out the account that the Number of Task-Rules manipulation influenced response coding.

## General discussion

The results from three experiments show that increasing the number of novel arbitrary rules held in working memory influences instructions-based performance (RITL). Importantly, increasing the number of familiar rules that hypothetically should not (or minimally) rely on WM resources, did not influence RITL thus pointing to the unique contribution of WM. In contrast, increasing WM load did not differentially influence the automaticity of instructions (manifested in the NEXT compatibility effect), suggesting a dissociation between RITL performance and its associated automaticity effect. The results regarding RITL and automaticity are further discussed separately, followed by broader theoretical implications.

### Rapid instructed task learning is uniquely influenced by working memory load

The current findings concerning RITL support previous hypotheses suggesting that the ability to perform actions immediately following instructions depends on available WM resources [8,20]. One such resource may be the “bridge” described by Oberauer [12], since novel S-R rules are hypothesized to be held in it. This finding also fits Ruge et al.’s [19] results regarding the influence of WM load on instructions-based performance using a different WM manipulation.

A broader issue, although not specifically tested in this study, concerns individual differences in WM capacity and RITL. The results demonstrate that conclusions drawn from individual differences studies do not hold in this experimental study. While the current results seem to be discrepant with the individual differences results found by Meiran et al. [18] and Ruge et al. [19], who showed that WM capacity was not significantly correlated with RITL; they are in-line with Ruge et al.’s findings regarding the influence of WM load on instructions-based learning. Therefore, future research focusing on this issue could perhaps both manipulate set-size and incorporate simple and complex WM spans in order to try and clear the picture regarding the complex relations between RITL, WM, and their individual differences.

### Automatic effects of instructions are not differentially influenced by working memory load

The predictions regarding automatic effects of instructions were not as clear as they were for RITL. Accordingly perhaps, the results were not as straightforward as one might hope for. We found a non-specific influence of WM load on the NEXT phase, showing a general increase in NEXT RT, that did not significantly modulate the NEXT compatibility effect. The fact that the NEXT effect was not modulated by WM load suggests a dissociation with respect to RITL, but the main effect of load on NEXT RT suggests some sensitivity to WM load. Importantly, while the NEXT effect is related to the novel rules which were instructed at the beginning of the mini-block, NEXT RT is not related to these rules. It reflects the operation of a repetitive rule that is relevant for the entire experiment: if the stimulus appears in red color → press NEXT. Thus, the slowing of NEXT responses may be irrelevant to the automaticity of the newly instructed rules–which is relatively insensitive to WM-load.

On the one hand, these results contradict those of Cohen-Kdoshay and Meiran [23] and Meiran and Cohen-Kdoshay [26], who showed that the first-trial flanker effect was diminished under WM load, manipulated by adding novel rules for a *secondary* task (whereas we loaded the main task directly). While the different results could be due to the different manipulation of load, they might also reflect the fact that the first-trial flanker effect reflects semantic priming, an account that does not hold for the NEXT compatibility effect that we used as our index of automaticity.

To sum up, the present results point to a dissociation between RITL and automatic effects of instructions with respect to WM load. These results correspond to a recent training study, showing that RITL (but not the NEXT compatibility effect) was influenced by WM training [29]. Broader theoretical implication concerning this dissociation are discussed below.

### Theoretical implications

The NEXT compatibility effect is considered to reflect a reflexive activation of *instructions*. Given that the results clearly demonstrate that loading WM with novel instructions harms the efficient implementation of the rules, it is puzzling how the NEXT compatibility effect remains uninfluenced by WM load. This dissociation could be accounted for by a recent model [8] Specifically, Cole et al. suggested a mechanism termed “task representation buffer”, which temporarily holds the instructions towards performance in the GO phase, shielding them until the interference caused by the NEXT phase is over. The current results could perhaps be taken as support for this hypothesis.

According to Cole et al. [8], the buffer is hypothetically located in the anterior prefrontal cortex, and it supports the hierarchical representation of the task which is required due to the task structure. Specifically, to ensure efficient performance in both the NEXT and GO phases, participants should hold a task representation stating that, “*If* RED color → press right(/left), *else if* GREEN color → [if X → right, and if Y → left]” (and this representation should be further elaborated for a higher number of bindings). This type of task representation is considered to involve “branching control” [39], since executing the task demands more than simply identifying target identity, as in standard choice tasks (i.e., deciding on the “branch” depending on the target color). Therefore, Cole et al. suggested that, in the NEXT paradigm, an effective strategy to manage these control demands would be to temporarily store the GO instructions in a buffer in the anterior prefrontal cortex, until their implementation, when their representation is fully activated.

The task-buffer perfectly accounts for the current results, as it suggests that during the ongoing NEXT task performance, the novel instructions are not yet held in the lateral prefrontal cortex (where they are connected to motor areas), *regardless* of the number of novel bindings. Importantly, as noted by Cole et al. [8], it also explains the individual differences that were demonstrated by Meiran et al. [18] showing a negative correlation between efficient RITL and small compatibility effects; such that individuals with an efficient buffer both benefit by protecting the novel instructions and show smaller reflexive activation of the instructions. The effect of WM load on NEXT RT could be taken to reflect a detrimental influence of holding an increased number of instructions when the NEXT phase is executed. This suggestion does not contradict the task-buffer model, since NEXT RT (as opposed to the NEXT compatibility effect) is unrelated to the specific content to the buffer, suggesting that the content is indeed “buffered”.

### Limitations

Probably, the most serious limitation is that the GO trial effect was reversed in Experiment 3 under the 6 task-rules conditions in both the novel- and familiar-rules-load groups. While this effect suggests that we should not examine whether the *GO trial effect* was influenced by WM load, we nonetheless do not fully understand what caused this reversal. On the one hand, it could be the motor complexity involved in this experiment; while, on the other hand, it could be the increased number of rules (six instead of four bindings, that even in the familiar-rules-load group they were not as familiar as the arrows of Experiment 2). Whereas this effect does not undermine our main conclusion, it will still be interesting to examine in future research how RITL is influenced by these issues.

Second, in the current study, we only increased the WM load involved with RITL, and showed a dissociation by which RITL, but not its associated automaticity, was affected. It is possible that loading the ongoing NEXT task would produce different results.

### Conclusion

The current study demonstrates a dissociation showing that the ability to perform tasks immediately following instructions specifically relies on available WM resources, but that its associated automaticity does not. These results offer support for the “task-buffer” hypothesis [8] which suggests that novel instructions are “set-aside” until their implementation in situations involving deferred performance.

## Supporting information

S1 Appendix General RT and the NEXT compatibility effect. Delta plot demonstrating the NEXT compatibility effect as a function of general RT.
(DOCX)
