# Supplementary material for: Rapid instructed task learning (but not automatic effects of instructions) is influenced by working memory load
Source: PLoS One. 2019 Jun 6;14(6):e0217681. doi: 10.1371/journal.pone.0217681 (PMC6553735; doi:10.1371/journal.pone.0217681)
Supplement: S1 Appendix — Delta plot demonstrating the NEXT compatibility effect as a function of general RT. (DOCX) [file pone.0217681.s001.docx]

**S1 Appendix**

**General RT and the NEXT compatibility effect**

In order to test whether the first NEXT trial shows a larger NEXT compatibility effect regardless of general RT, we re-analyzed previous results ([1] Experiment 1). We divided RT per condition (NEXT Trial (1-5), and Compatibility (compatible-incompatible)) into three percentiles: 0-0.33, 0.33-0.66, and 0.66-1 (since the task involves a relatively small number of trials per condition, we only used three percentiles). NEXT trials 4 and 5 were pooled together in order to avoid missing data.

The results are presented in a delta plot [2,3], which shows the NEXT compatibility effect as a function of general RT (Fig S1). This allowed us to test whether the effect changes with response slowing. NEXT trials were inserted separately, and the plot demonstrates similar RT (350-450 ms), and that the NEXT compatibility effect is not larger in the first trial relative to the more advanced trials. Nonetheless, it seems that the NEXT compatibility effect increases with RT, and since the first trial sometimes produces longer RT, it shows the NEXT compatibility effect to a greater degree.


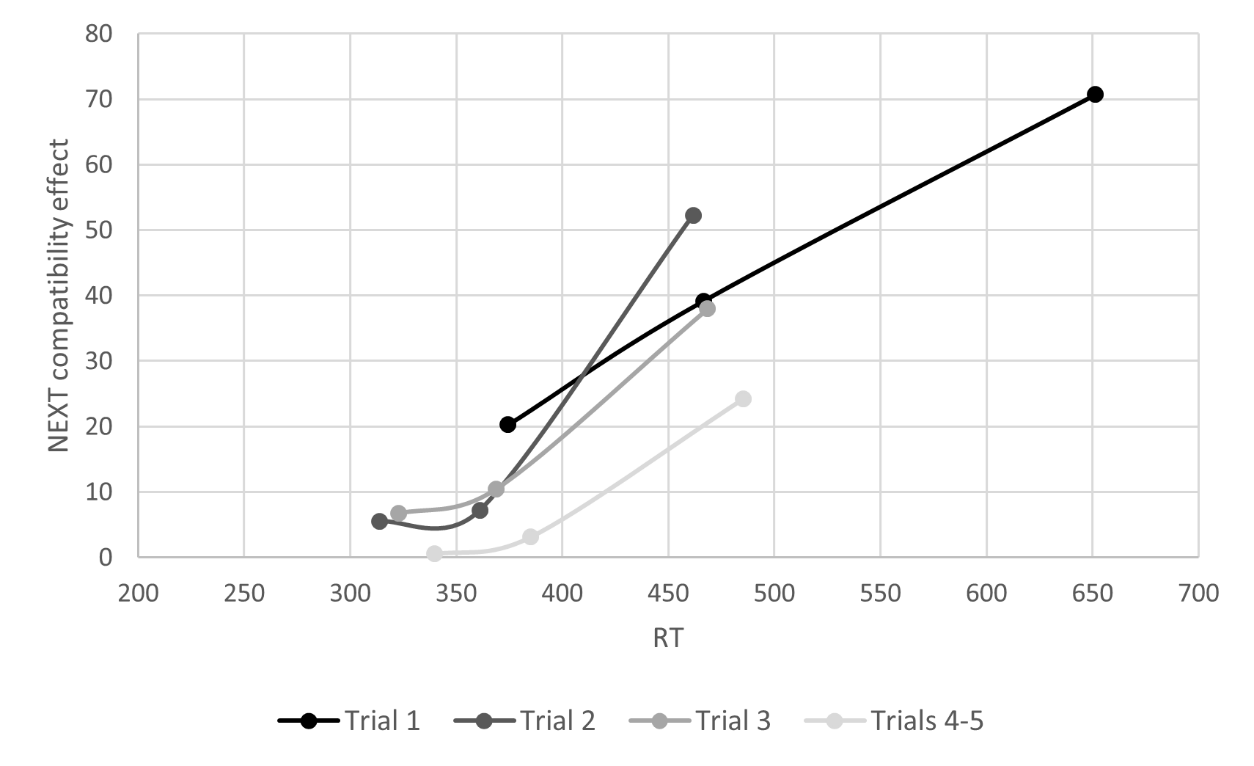


**Fig S1. Delta plot demonstrating the NEXT compatibility effect as a function of general RT**. (data reanalyzed from Meiran et al., [1], Experiment 1).

**References**

1. Meiran N, Pereg M, Kessler Y, Cole MW, Braver TS. The power of instructions: Proactive configuration of stimulus–response translation. J Exp Psychol Learn Mem Cogn. 2015;41(3): 768–786.

2. De Jong R, Liang C-C, Lauber E. Conditional and unconditional automaticity: A dual-process model of effects of spatial stimulus-response correspondence. J Exp Psychol Hum Percept Perform. 1994;20(4): 731–750.

3. Ridderinkhof RK. Micro- and macro-adjustments of task set: Activation and suppression in conflict tasks. Psychol Res. 2002;66(4): 312–323.
